# P103 VRP-034 at higher doses: a comprehensive preclinical evaluation of polymyxin B-associated nephrotoxicity across *in vitro*, *in vivo* and translational models

**DOI:** 10.1093/jacamr/dlaf230.110

**Published:** 2025-12-04

**Authors:** Kamlesh Vishwakarma, Anurag Payasi, Saransh Chaudhary, Anmol Aggarwal

**Affiliations:** Venus Medicine Research Centre, India; Venus Medicine Research Centre, India; Venus Medicine Research Centre, India; Venus Medicine Research Centre, India

## Abstract

**Background:**

Polymyxin B (PMB) is a last-line antibiotic for MDR Gram-negative infections, but its clinical use is hampered by dose-limiting nephrotoxicity. VRP-034, a supramolecular cationic (SMC) formulation of polymyxin B, was developed to mitigate renal toxicity. This study synthesizes findings from a comprehensive preclinical program to compare the nephrotoxicity of high-dose VRP-034 against lower-dose marketed PMB across a spectrum of *in vitro*, *in vivo* and translational models.

**Methods:**

Data were synthesized from fourteen studies utilizing cytotoxicity assays (HK-2, primary hPTEC), advanced 3D human kidney-on-a-chip platforms and *in vivo* animal models. Nephrotoxicity was assessed using FDA-qualified biomarkers (KIM-1, NGAL, cystatin C, clusterin, osteopontin, NAG), oxidative stress and inflammation markers, and renal histopathology. In vivo, VRP-034 (18–24 mg/kg/day) was compared to marketed PMB (12–18 mg/kg/day). Pharmacokinetic (PK) profiles were evaluated for both formulations.

**Results:**

VRP-034 demonstrated significantly reduced nephrotoxicity across all models, even at higher doses. In cytotoxicity assay (HK-2 cells), VRP-034’s IC₅₀ was 147 μg/mL versus 90.4 μg/mL at 24h and 86.3 μg/mL versus 48.5 μg/mL at 48 h (*P<*0.0001). In primary hPTEC, VRP-034 reduced KIM-1 by ∼3-fold and NGAL/clusterin by ∼2-fold at 10–50 µM. In the 3D kidney-on-a-chip model, VRP-034 preserved tubular architecture and reduced key biomarkers (KIM-1, cystatin C, clusterin, osteopontin) by up to 70%, while limiting pro-inflammatory cytokines and apoptosis markers. In animals, it reduced early kidney injury biomarkers by ≥50% compared to marketed PMB and caused only mild, reversible histopathological changes, unlike marketed PMB, which induced severe tubular damage. Crucially, this improved safety was not attributable to altered drug exposure, as no significant differences in key PK parameters (AUC_0–24_, C_max_, *t*_½_) were observed between the two formulations.

**Conclusions:**

Despite higher administered doses, VRP-034 consistently demonstrated a superior renal safety profile compared to marketed PMB, a finding supported by robust data across a comprehensive suite of preclinical models. The preserved pharmacokinetic profile confirms that the safety benefit is intrinsic to the formulation. VRP-034 shows strong clinical potential to overcome the dose-limiting nephrotoxicity of polymyxins, enabling safer and more effective treatment of MDR Gram-negative infections.

